# Development and Evaluation of Monoclonal Antibodies for the Glucoside of T-2 Toxin (T2-Glc)

**DOI:** 10.3390/toxins5071299

**Published:** 2013-07-18

**Authors:** Chris M. Maragos, Cletus Kurtzman, Mark Busman, Neil Price, Susan McCormick

**Affiliations:** 1 Bacterial Foodborne Pathogens and Mycology Research Unit, USDA-ARS-NCAUR, 1815 N. University St., Peoria, IL 61604, USA; E-Mails: cletus.kurtzman@ars.usda.gov (C.K.); mark.busman@ars.usda.gov (M.B.); susan.mccormick@ars.usda.gov (S.M.); 2 Renewable Product Technology Research Unit, USDA-ARS-NCAUR, 1815 N. University St., Peoria, IL 61604, USA; E-Mail: neil.price@ars.usda.gov

**Keywords:** T-2 toxin, masked mycotoxins, metabolites, antibody, immunoassay

## Abstract

The interactions between fungi and plants can yield metabolites that are toxic in animal systems. Certain fungi are known to produce sesquiterpenoid trichothecenes, such as T-2 toxin, that are biotransformed by several mechanisms including glucosylation. The glucosylated forms have been found in grain and are of interest as potential reservoirs of T-2 toxin that are not detected by many analytical methods. Hence the glucosides of trichothecenes are often termed “masked” mycotoxins. The glucoside of T-2 toxin (T2-Glc) was linked to keyhole limpet hemocyanin and used to produce antibodies in mice. Ten monoclonal antibody (Mab)-producing hybridoma cell lines were developed. The Mabs were used in immunoassays to detect T2-Glc and T-2 toxin, with midpoints of inhibition curves (IC_50_s) in the low ng/mL range. Most of the Mabs demonstrated good cross-reactivity to T-2 toxin, with lower recognition of HT-2 toxin. One of the clones (2-13) was further characterized with in-depth cross-reactivity and solvent tolerance studies. Results suggest Mab 2-13 will be useful for the simultaneous detection of T-2 toxin and T2-Glc.

## 1. Introduction

**Figure 1:**
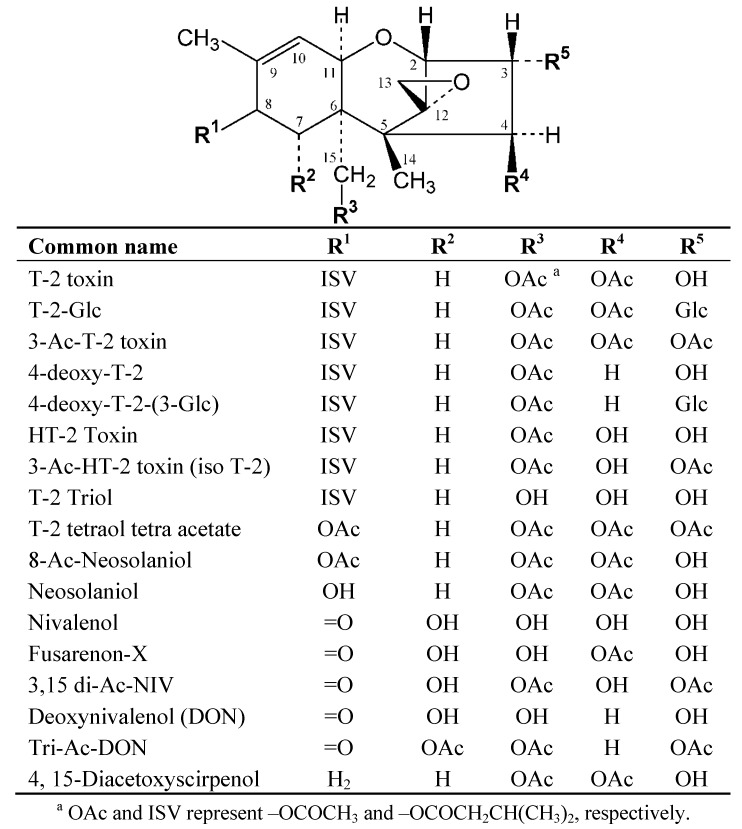
Structures of certain trichothecene toxins.

Various species of fungi routinely cause disease in important cereal crops. Among these, several species of *Fusarium* infect wheat, maize, oats, barley, and rice. In addition to the loss of value resulting from lowered food quality, the fungi may produce certain secondary metabolites, mycotoxins, which are harmful to animals and humans. T-2 toxin is one of a group of trichothecene mycotoxins produced by *F. sporotrichioides*, *F. poae*, and *F. langsethiae*. A related toxin, HT-2 toxin (Figure 1), is believed to be formed by the deacetylation of T-2 toxin during metabolism or bioconversion by microflora.

Both toxins are routinely found in commodities, in particular oats. T-2 toxin is believed to act by inhibiting both protein and DNA synthesis and is acutely toxic to many species, including mammals [1]. While poultry are, in general, more resistant to the effects of mycotoxins they are nevertheless susceptible to oral necrosis, asthenia, inappetence, diarrhea, decreased growth rate, decreased egg production, thinner egg shells, and immune-system toxicity of T-2 toxin [2,3,4]. Poultry can also exhibit neurological effects following exposure to T-2 toxin [4]. The Joint FAO/WHO Expert Committee on Food Additives (JECFA) and the European Food Safety Authority (EFSA) have published opinions on the risks posed by T-2 and HT-2 toxins in foods [2,5]. The need to monitor for trichothecenes in commodities and foods has instigated the development of many different methods for their detection. The literature on analysis of T-2 toxin and related trichothecenes has been recently reviewed [6,7,8]. Techniques that have been developed to detect T-2 toxin include instrument-intensive methods such as liquid chromatography (LC) with diode array detection [9], labeling and fluorescence detection [10], or mass spectrometric detection [11]. To facilitate sample cleanup or more rapid analysis by immunoassay, numerous antibodies have been developed against T-2 and HT-2 toxins [12,13,14,15,16,17,18,19,20,21,22,23,24,25,26,27,28]. In addition to enzyme-linked immunosorbent assays (ELISAs), immunoassays for T-2 toxin include radioimmunoassay [12,16,20,21], lateral flow test strips [26], fluorescence polarization immunoassay [29], an optical immunoassay [30], and an immunochip [31].

Animals and plants alike have mechanisms to metabolize trichothecene mycotoxins, presumably to render them less toxic or facilitate their elimination. The structure of T-2 toxin (Figure 1) reveals several sites for potential metabolism. In addition to deacetylation, other mechanisms involve deacylation and de-epoxidation. Furthermore the parent toxins and their metabolites may be acted upon by phase II metabolism, yielding a variety of conjugated products. The study of the metabolites of mycotoxin exposure in animals (e.g., biomarkers) is intertwined with the study of metabolites of mycotoxins in the fungi and the host plants. In the latter case the products are known colloquially as “hidden” or “masked” mycotoxins. The term is intended to imply that they are poorly detected, or not detected at all, using conventional methods for measuring the parent toxins. There is considerable interest in the possibility of such metabolites serving as reservoirs of the parent toxins, and a recent issue of the World Mycotoxin Journal focused on this subject [32]. Because of the large number of mycotoxins and possibilities for their metabolism, the number of known masked mycotoxins continues to expand, as such, analytical methods for their detection continue to be developed. A biomarker of exposure in one plant or animal system might be a masked toxin for another system; hence “metabolite”, “biomarker”, and “masked mycotoxin” are not mutually exclusive. Despite such ambiguities an understanding of the formation of such metabolites and their role as potential reservoirs of toxicity is important. 

In the case of the trichothecene mycotoxins, several masked mycotoxins have been reported. These have included the glycosylated derivatives of DON [11,33,34]. In particular the 3-glucoside of DON (DON-3-Glc) has been widely described in the literature. DON-3-Glc is one of the very few masked mycotoxins for which analytical standards are commercially available. Commercial immunoassays developed to detect DON have also been shown to detect the DON-3-Glc to varying degrees [16,35,36,37]. Recently several reports have appeared describing analogous glucosides and glucuronides in the group A trichothecenes that include T-2 and HT-2 toxins [38,39,40,41,42]. The modification of T-2 toxin by yeasts, yielding T-2 toxin with glucose attached at the C-3 position (e.g., T2-Glc) was recently reported [43]. To facilitate the detection of T2-Glc, we endeavored to develop an antibody capable of interacting with this metabolite. While cross-reaction of antibodies with masked mycotoxins, in particular DON-3-Glc, has been demonstrated [37], to our knowledge ours is the first report of an antibody developed specifically against a masked mycotoxin.

## 2. Results and Discussion

### 2.1. Production of Mabs to T2-Glc

T2-Glc was conjugated to two proteins, OVA and KLH. The T2G-KLH was used to immunize mice, while the T2G-OVA was used as the immobilized antigen for evaluation of potential antibodies. The attachment of T2-Glc to OVA in the T2G-OVA conjugate was evaluated by ESI-MS. To obtain information on the mass and glycosylation heterogeneity of the intact glycoprotein, mass spectra were first acquired of OVA. The full-scan spectrum was obtained and the resulting deconvoluted spectra displayed a series of mass additions to the molecular weight of OVA with a combination of 162 and 203 Da mass increments, corresponding to hexose and *N*-acetylglucosamine residues. The only glycosylation site of ovalbumin (Asn-292) has been observed to possess a variety of non-sialylated oligosaccharide structures. The mass and degrees of glycosylation of OVA were consistent with observations recorded by Saba *et al*. [44]. Similarly, ESI mass spectra were acquired of T2G-OVA. The deconvoluted spectra indicated a positive shift of the envelope of the observed masses of the T2G-OVA from those observed for OVA by approximately 1300 Da. Given that conjugation with one T2-Glc would be expected to increase protein mass by 656 Da, such a shift suggests that, on the average, two T2-Glc were added to each OVA during the conjugation. As such the T2-OVA was expected to be suitable as an immobilized antigen for evaluation of possible T2-Glc antibodies.

The T2G-KLH conjugate was used to immunize 10 mice, all of which developed a serum response to T2-Glc as measured by competitive indirect (CI)-ELISA. Subsequently, two of the mice were used in hybridoma fusions that yielded a total of 53 positive cultures having antibodies that bound T2-Glc. From these10 clones were subsequently isolated and used to produce sufficient antibody for further evaluation. The Mabs were tested for activity against three toxins (T2-Glc, T-2 toxin, HT-2 toxin) by CI-ELISA (Table 1). 

**Table 1:** Response of 10 Mabs to T2-Glc, T-2 toxin, and HT-2 toxin.

	IC_50_ (ng/mL) ^a^				Cross-reactivity (%) ^b^	
Mab	T2-Glc	T-2 toxin	HT-2 toxin	Mab	T-2 toxin	HT-2 toxin
1-2	623 ± 15	614 ± 47	>60000	1-2	101 ± 8	<1.0
1-3	8.6 ± 0.8	6.3 ± 0.6	43.2 ± 7.2	1-3	135 ± 14	19.9 ± 3.3
1-4	13.3 ± 1.7	13.5 ± 0.6	243 ± 22	1-4	99.0 ± 5.0	5.5 ± 0.5
2-5	13.4 ± 0.6	14.0 ± 1.9	33.8 ± 2.8	2-5	96.3 ± 13.1	39.8 ± 3.3
2-11	17.6 ± 0.7	20.2 ± 1.2	562 ± 71	2-11	86.9 ± 5.5	3.1 ± 0.4
2-13	3.5 ± 0.4	3.8 ± 0.1	271 ± 19	2-13	91.6 ± 4.1	1.3 ± 0.1
2-16	13.6 ± 0.3	17.4 ± 1.0	321 ± 16	2-16	78.6 ± 4.9	4.3 ± 0.2
2-17	11.3 ± 0.1	16.7 ± 1.3	118 ± 12	2-17	67.5 ± 5.5	9.5 ± 0.9
2-21	28.2 ± 0.5	23.1 ± 1.0	255 ± 4	2-21	122 ± 5	11.1 ± 0.2
2-44	7.8 ± 0.4	8.5 ± 0.2	28.1 ± 5.4	2-44	91.3 ± 2.6	27.7 ± 5.3

^a^ Concentration of compound that inhibited binding of the antibody by 50% in CI-ELISA. Values shown were derived from triplicate plates for each antibody and indicate the mean ± 1 standard deviation; ^b^ Percentage cross reactivity relative to T2-Glc. Cross reaction was calculated as [(IC_50_ of T2-Glc/IC_50_ of analog) × 100%].

Of the antibodies, the Mab from clone 2-13 was the most sensitive to T2-Glc and was also the most sensitive to T-2 toxin. Interestingly, half of the clones (1-2, 1-4, 2-5, 2-11, 2-13, 2-44) were almost equally cross-reactive to T-2 toxin as they were to T2-Glc. Such antibodies would be good for assays where both metabolites need to be measured. Two of the Mab showed better binding for T-2 toxin than for T2-Glc. However, even in those cases (Mab 1-3, 2-21) the responses to the two toxins were fairly close, with the greatest cross-reactivity being only 135% (Mab 1-3). Taken in the context of all 10 antibodies this implies that the glucose portion of the immunogen (T2G-KLH) was not a major contributor to antibody binding. It further suggests that the glucose portion of the molecule acted predominantly as a linker between the T-2 toxin and the protein, and was not a focal point for the immune response. 

T-2 toxin and HT-2 toxin have similar structures, but differ in that T-2 toxin has an acetate at the C-4 position that is absent in HT-2 toxin (Figure 1). The data in Table 1 reveal that none of the antibodies recognized HT-2 toxin better than T-2 toxin or T2-Glc. Eight of the 10 Mabs demonstrated cross-reactivities to HT-2 toxin of less than 20% (Table 1). Mab 2-5, with a relative activity of approximately 40% was the most cross-reactive to HT-2 toxin. The relatively poorer recognition of HT-2 toxin and good recognition of T-2 toxin demonstrated by most of the antibodies, suggests that the acetate at the C4 position of the trichothecene was an important contributor to the antibody binding and likely an important contributor to the immune response. 

### 2.2. Cross-Reactivity of Mab 2-13 with Additional Trichothecenes

From the results in Table 1 it was clear that one of the antibodies (2-13) was more sensitive for T2-Glc and T-2 toxin than the others. In additional assays the Mab 2-13 demonstrated an IC_50_ of 3.25 ng/mL of T-2 toxin, which was not significantly different from the 3.5 ng/mL reported in Table 1. Because 0.05 mL was used in each assay, this was equivalent to an IC_50_ of 162 pg T-2 toxin per assay. There is considerable literature on the development of immunoassays for T-2 toxin [12,13,14,15,16,17,18,19,20,21,22,23,24,25,26,27,28]. Unfortunately not all publications use the same metrics for reporting upon assay sensitivity. However, many reports have given some indication of IC_50_ either in the form of amount per assay (that is pg T-2 toxin/assay) or concentration (that is ng/mL or pg/mL). In certain cases the values have not been reported but can be roughly estimated from dose-response figures within the publication. Of the published immunoassays for T-2 toxin, three appeared notably more sensitive than the others [22,23,28]. Remarkably, one assay was reported to have an IC_50_ of 20 pg/mL [23]. Many reports have yielded antibodies with similar IC_50_s as the Mab 2-13 reported here, that is, in the range of 1–5 ng/mL or circa 0.2 ng/assay [13,15,16,20,25,27]. Assays with sensitivities poorer than these have also been commonly reported. Taken within the context of the existing literature, the CI-ELISA reported here using Mab 2-13, is neither remarkably better nor worse than previous immunoassays for T-2 toxin. If the Mab 2-13 has an advantage it may be in the ability to bind and detect the T2-Glc. Given our results, it would be of interest to determine if immunoassays previously developed for T-2 toxin might also recognize T2-Glc.

In order to determine which portions of the trichothecene molecule were most important for antibody binding, Mab 2-13 was tested against an additional 14 trichothecenes (Table 2). 

**Table 2:** Cross-reactivities of 17 trichothecenes with Mab 2-13 in PBS.

Common name	Cross-Reactivity to Mab 2-13 (%)	^a^ R^1^	R^2^	R^3^	R^4^	R^5^
T-2-Glc	100	ISV	H	OAc	OAc	Glc
T-2 toxin	91.6 ± 4.1	ISV	H	OAc	OAc	OH
4-deoxy-T-2-(3-Glc)	2.6 ± 0.1	ISV	H	OAc	H	Glc
8-Ac-Neosolaniol	2.5 ± 0.2	OAc	H	OAc	OAc	OH
3-Ac-T-2 toxin	2.3 ± 0.1	ISV	H	OAc	OAc	OAc
HT-2 Toxin	1.3 ± 0.1	ISV	H	OAc	OH	OH
3-Ac-HT-2 toxin (iso T-2)	0.1 to 1	ISV	H	OAc	OH	OAc
4-deoxy-T-2	0.1 to 1	ISV	H	OAc	H	OH
Neosolaniol	0.1 to 1	OH	H	OAc	OAc	OH
T-2 Triol	<0.1	ISV	H	OH	OH	OH
T-2 tetraol tetra acetate	<0.1	OAc	H	OAc	OAc	OAc
Nivalenol (NIV)	<0.1	=O	OH	OH	OH	OH
Fusarenon-X	<0.1	=O	OH	OH	OAc	OH
3,15 di-Ac-NIV	<0.1	=O	OH	OAc	OH	OAc
Deoxynivalenol (DON)	<0.1	=O	OH	OH	H	OH
Tri-Ac-DON	<0.1	=O	OAc	OAc	H	OAc
4,15-Diacetoxyscirpenol	<0.1	H_2_	H	OAc	OAc	OH

^a^ R groups refer to the generic trichothecene structure in Figure 1.

The data suggest that this Mab is highly specific for T-2 toxin and T2-Glc, with poor cross-reactivity for the remaining 15 trichothecenes. However, there were patterns within the data that suggest certain portions of the trichothecene structure were more important to binding than others. An examination of Table 2 reveals that, of the six toxins that cross-reacted more than 1% with the antibody, only one (8-acetyl-neosolaniol) lacked an isovaleryl group at C-8 (R1 in Figure 1). All six of the most cross-reactive toxins were also reduced at C-7 (R2 in Figure 1) and contained an acetate at C-15 (R3 in Figure 1). The impact of changes to functional groups at positions 8, 7, and 15 on the trichothecene backbone, suggest the importance of this region for recognition by Mab 2-13. However, that was not the only region important for selectivity. As described previously with regard to the cross reactivity of all 10 Mabs to HT-2: the absence of an acetate at position 4 (R4 in Figure 1) significantly reduced binding. The importance of an acetate at position 4 was also revealed by the poor cross-reactivity of 4-deoxy-T2-Glc, which differed from T2-Glc only at R4. The antibody binding site was better able to accommodate certain changes to position 3 (R5 in Figure 1). Substitution of the glucose with a hydroxyl group did not substantially reduce binding, as evidenced by the high cross-reactivity of T-2 toxin. However, substitution with an acetate (e.g., 3-Ac-T2) significantly reduced binding. 

These data suggest there may be a certain amount of flexibility allowed for binding at R5. This may be important for future use of the Mab 2-13. The isomer of T2-Glc used to make the immunogen was the same isomer as that produced by yeast cultures (*i.e.*, linked by an axial, or α, glycosidic bond). While the α-isomer of T2-Glc was naturally produced by the yeast, the identity of the isomers of T2-Glc present in naturally contaminated commodities are unknown. Previous reports with the glucoside of the related trichothecene DON have suggested that when acted upon by UDP-glucosyltransferase from *Arabidopsis thaliana*, the product is the β isomer, that is, 3-β-D-glucopyranosyl-4-DON [45]. Given the presence of at least some flexibility of the antibody for changes to the R5 position, it may bind to the β form as well. Unfortunately, the lack of an available standard of β-linked T2-Glc prevented us from testing it for cross-reactivity. Ultimately it will be important to determine which isomer is present in naturally contaminated grains. The need for further research into the cross-reactivity of antibodies to the glycosylated trichothecenes is further supported by the recent discovery of oligoglycosides of DON [46]. It is not difficult to speculate that similar oligoglycoside conjugates with T-2 toxin may also exist in T-2 contaminated foods. 

### 2.3. Solvent Tolerance

Methods of analysis for T-2 toxin generally involve an extraction of the toxin from grains using mixtures of methanol or acetontrile with water. Because of the relatively hydrophobic nature of T-2 toxin such mixtures tend to have a high solvent content. Addition of the glucose (*i.e.*, to T2-Glc) increases the polarity of toxin. For these reasons it was important to test the Mab 2-13 for tolerance to multiple levels of solvents. T2-Glc standards were prepared in aqueous solutions of up to 50% (by volume) methanol or up to 30% acetonitrile and tested by CI-ELISA. There was a gradual deterioration in assay performance with increased solvent content (Table 3).

**Table 3:** Effect of Solvents on the Response of Mab 2-13 to T2-Glc.

Solvent	Solvent concentration ^a^	IC_50_ (ng/mL) ^b^	Relative response (%) ^c^	*N* ^d^
PBS	0	3.3 ± 0.1	100%	12
Methanol	5%	3.3 ± 0.2	98%	3
	10%	3.7 ± 0.1	87%	3
	20%	4.0 ± 0.3	81%	12
	30%	4.9 ± 0.5	66%	3
	50%	9.2 ± 0.9	35%	3
Acetonitrile	5%	3.7 ± 0.2	87%	3
	10%	4.4 ± 0.3	73%	6
	15%	6.3 ± 0.2	52%	3
	20%	7.4 ± 0.2	44%	3
	30%	14.3 ± 1.5	23%	3

^a^ Volume percentage of the solvent used to prepare the standards. Because the standards were mixed (1 + 1) with Mab in the assays, the concentration of solvent present during the competitive step was one half of the concentrations listed; ^b^ Average IC_50_ ± 1 standard deviation for T2-Glc; ^c^ Response of T2-Glc in indicated solution relative to T2-Glc in PBS (100%). Calculated as (IC_50_ in PBS)/(IC_50_ in solvent solution) × 100%; ^d^ Number of ELISA plates used to determine the summary statistics listed.

**Figure 2:**
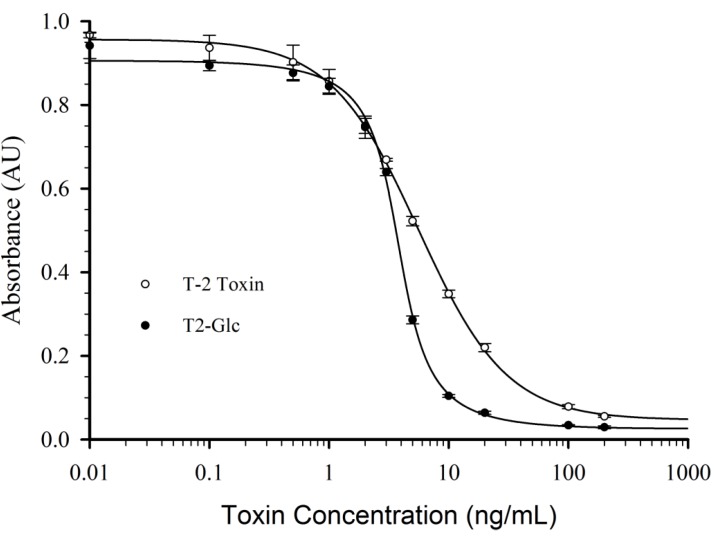
Response of the Mab 2-13 CI-ELISA to T-2 and T2-Glc standard solutions in 20% methanol. T-2 data were fit with a logistic dose-response curve, T2-Glc data were fit with a Lorentzian Cummulative curve. Data are averages from three plates ± 1 standard deviation.

The solvent concentration at which the IC_50_ doubled was between 30% and 50% for methanol, and approximately 20% for acetonitrile. Therefore the assay was more easily disrupted with acetonitrile than with methanol. Comparison of solvent tolerance of Mab 2-13 with previous T-2 antibodies is difficult, because most of the previous literature reports did not include such data. Baumgartner *et al*. [27] developed an assay with an IC_50_ for T-2 toxin of 3.2 ng/mL, which was very similar in sensitivity to that of our assay using Mab 2-13. That report demonstrated that 10% methanol shifted the response curve to higher concentrations. The assay could be used at up to 10% methanol, but at 15% the antibody was not able to work. As with our Mab 2-13, the assays were more sensitive to disruption by acetonitrile than methanol [27]. In another case, using a sensitive competitive direct ELISA for T-2 toxin [25], both acetonitrile and ethanol inhibited binding of toxin-peroxidase conjugate to the immobilized antibody. The effect could be avoided by using solvent concentrations of less than 10%. Another report incorporated 10% methanol during the cross-reactivity studies [19], implying that the assay was able to tolerate at least that level. To determine whether methanol would impact cross-reactivity of the Mab 2-13 assay, the response to T-2 toxin was also tested in 20% methanol (Figure 2).

When T-2 toxin was tested in PBS the cross-reactivity was 92% relative to T2-Glc (e.g., Table 2). However, when T-2 toxin was tested in 20% methanol, the cross-reactivity was 63% relative to T2-Glc (Figure 2). This suggests that the relative cross-reactivity will be influenced by the percentage of methanol present. Therefore if Mab 2-13 is to be used to detect T-2 toxin or T2-Glc in commodities, the cross-reactivity should be determined under the solvent conditions specific to such assays. The response curve of T2-Glc in 20% methanol had a very steep slope between 1 and 10 ng/mL, which may be very useful in an assay based upon signal threshold rather than quantitation. Within the context of these results, and the literature, it appears that the assay with our Mab 2-13 has relatively good solvent tolerance. This, combined with the ability to equally detect T-2 toxin and T2-Glc, may make this antibody useful for the simultaneous detection of T-2 toxin and its 3-glucoside in samples of commodities.

## 3. Experimental Section

### 3.1. Reagents

Chicken egg albumin (OVA), polyvinyl alcohol (PVA, average molecular weight 30,000 to 70,000), and 1,1'-carbonyldiimidazole (CDI) were purchased from Sigma-Aldrich (Milwaukee, WI, USA). Keyhole limpet hemocyanin (KLH) was purchased from Pierce Chemical (Rockford, IL, USA). Peroxidase conjugated goat anti-mouse IgG was purchased from Jackson ImmunoResearch Laboratories, Inc. (West Grove, PA, USA). T-2 toxin and HT-2 toxin were obtained from Sigma-Aldrich. The following trichothecenes were prepared at the National Center for Agricultural Utilization Research (NCAUR; Peoria, IL, USA) by one of the co-authors (S.M.) from *Fusarium* cultures: deoxy-T2, iso-T-2 toxin, 3-Ac-T2, T-2 triol, TTTA, NEO, 8-Ac-NEO, Tri-Ac-DON, FX, 3,15- diAc-NIV, and DAS. T2-Glc and deoxy-T2-Glc were produced at NCAUR by incorporating T-2 toxin or 4-deoxy T-2 toxin into the culture medium for the yeast *Blastobotrys muscicola.* They were isolated as described previously [43]. Data from NMR indicated that the glucosidyl group was O-linked to the T-2 toxin by an axial (α-) glycosidic bond [43]. Stock solutions of T2-Glc were prepared by gravimetric methods followed by dilution in acetonitrile. 

### 3.2. HPLC with Photodiode Array Detection

**Figure 3:**
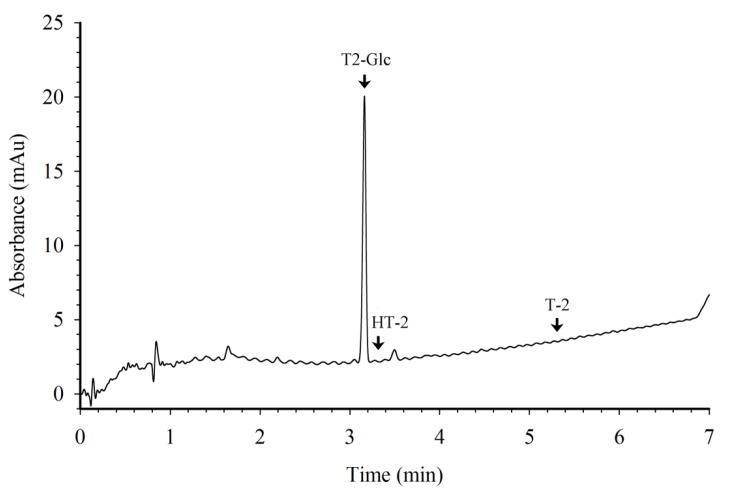
HPLC chromatogram of T2-Glc used to prepare the protein conjugates. The arrows indicate retention times for T2-Glc (3.17 min), HT-2 toxin (3.30 min), and T-2 toxin (5.35 min). The amount of T2-Glc injected was 250 ng.

The purity of the T-2 toxin, HT-2 toxin, and T2-Glc were also assessed by HPLC with photodiode array detection. The instrumentation consisted of a Dionex Ultimate 3000 System (Thermo Fisher, Pittsburgh, PA, USA). Solvent A was acetonitrile, solvent B was water. The column was a Phenomenex Kinetix C-18, 2.6 µm, 4.6 mm × 15 cm, equipped with a Phenomenex RP guard cartridge. The mobile phase was a gradient, with solvent A acetonitrile and solvent B water, as follows: equilibrate for 3.5 min with 30%A; inject sample; linear ramp from 30%A to 50%A over 6 min; linear ramp to 90%A over 1.5 min; hold at 90%A for 1.5 min; then return to the equilibration condition at the end of the run (e.g., at 9 min). Flow rate of 1.7 mL/min. The detector was programmed to scan the range from 190 to 300 nm, with monitoring at 202 nm, data collection rate 10 Hz. The volumes injected were 10 µL. A sample chromatogram is indicated in Figure 3.

### 3.3. Preparation and Evaluation of T2-Glc Protein Conjugates

Protein conjugates of T2-Glc were synthesized by linking the hydroxyl groups of the toxin to the primary amines of the proteins using a carbodiimide technique similar to that described previously for DON [47]. The immunogen was a conjugate of T2-Glc with KLH (T2G-KLH). On the day of the reaction 4 mg of T2-Glc was dissolved in 0.4 mL acetone, and 75 mg of CDI was added. The vessel was sealed and held at ambient temperature for 1 h, after which 0.05 mL of water was added, followed by 1 mL of KLH solution (20 mg in 0.1 M sodium bicarbonate buffer, pH 8.5). The mixture was incubated for 24 h at 4 °C and then dialyzed against five sequential changes of PBS to remove unbound T2-Glc. The T2G-KLH was diluted to 2 mg/mL with 0.1 M PBS, then freeze-dried and sent to Harlan Bioproducts for Science (Madison, Wisconsin, USA) for administration into mice. The test antigen, a conjugate of T2-Glc with ovalbumin (T2G-OVA) was prepared in a similar fashion. The T2G-OVA was evaluated by mass spectrometry to determine the degree of conjugation with T2-Glc. The mass spectrometer (MS) used was an Exactive-MS (Thermo Fisher Scientific, Waltham, MA, USA) equipped with an electrospray ionization (ESI) source. For all experiments the MS was operated in positive ionization mode. Samples were sprayed using a 4.00 kV needle voltage, and optimized parameters of the system for MS detection were: inlet capillary voltage: +90.0 V; tube lens voltage: +190 V; capillary temperature: 275C; skimmer voltage: +40.0 V. All analyses were conducted with an automated gain control setting of 5 × 10^5^, a resolution setting of 100,000 FWHM, a scan speed of 1 Hz and a mass scan range of 500–3000 Da. Aqueous solutions of OVA or T2G-OVA (1 mg/mL) were infused into the ESI source with a syringe pump at a flow rate of 5 μL/min. A 20 μL/min supplemental flow of 1 + 1 (*v*/*v*) methanol/water with 2% acetic acid, provided by a high performance liquid chromatography pump, was added to the syringe pump flow via a tee immediately prior to the ESI source.

### 3.4. Immunizations and Screening for T2-Glc Specific Antibodies by CI-ELISA

Immunization of mice and collection of sera were conducted at Harlan Bioproducts for Science (Madison, WI, USA). Ten female Balb/C mice were initially immunized by injection of 100 µg T2G-KLH per animal using the same procedures as described previously for production of fusarin antibodies [48]. A CI-ELISA was developed and used for screening of mouse sera and culture supernatants for the presence of antibodies. Each hybridoma culture supernant solution was tested in single replicates at a minimum of three dilutions, and positive cultures were re-assayed. For screening assays 0.1 ml of T2G-OVA, 1 μg/mL in 0.05 M sodium phosphate buffer (pH 7), was added to wells of polystyrene microtiter plates and allowed to attach overnight at 4 °C. After washing the coated plate twice with 0.32 mL Tween-PBS (0.02% *v*/*v* Tween-20 in 0.01 M PBS pH 7), 0.32 mL of PVA-PBS (1% *w*/*v* PVA in 0.01 M PBS) was added and allowed to incubate at ambient temperature for 2 h. During this incubation, test solutions were prepared. The test solutions consisted of 0.06 mL of toxin standard solutions (or PBS control) mixed with 0.06 mL of serum (or culture fluid) diluted in BSA-PBS (1% *w*/*v* BSA in 0.01 M PBS) in the wells of a polypropylene microwell plate (Corning Inc., Corning, NY, USA). The wells of the polystyrene (T2G-OVA coated) plate were washed twice with Tween-PBS and 0.1 mL of test solution was transferred into each well. After incubation at ambient temperature for 30 min the plate was washed three times and 0.1 mL of goat anti-mouse peroxidase conjugate (diluted 1:2000 in BSA-PBS) was added. The plate was incubated for 30 min at ambient temperature then washed four times before addition of 0.1 mL of the *O*-phenylenediamine (OPD) substrate. The OPD solution was prepared by combining 0.02 mL of 30% H_2_O_2_ and 20 mg OPD in 50 mL of citrate-phosphate buffer (0.05 M citrate, 0.1 M phosphate, pH 5.0). After 5 min at ambient temperature, the reaction was stopped by the addition of 0.1 mL of 1 N hydrochloric acid. Color development was determined by measuring the absorbance at 490 nm using a Synergy HT microplate reader (Bio-Tek, Winooski, VT, USA). 

### 3.5. Production and Purification of Monoclonal Antibodies

Two mice having sera with antibodies reactive to T2-Glc were sacrificed and aseptically splenectomized at Harlan Bioproducts for Science (Madison, WI, USA). Spleenocytes were chemically fused with Balb/C non-immunoglobulin secreting (NS-1) myeloma cells using polyethylene glycol. Fused cells were plated in HAT selection media. After 11 days, HAT resistant cultures were isolated and screened for anti-T2-Glc activity by CI-ELISA. The two fusions yielded a total of 53 positive cultures. From these 10 cultures were subsequently cloned, expanded and frozen. The cultures are named here as: 1-2, 1-3, 1-4, 2-5, 2-11, 2-13, 2-16, 2-17, 2-21, and 2-44. With this nomenclature the first digit describes the fusion and the second digit describes the hybridoma cell line derived from that fusion. All antibodies were isotype IgG_1_. The 10 cell lines were expanded to produce ascites fluid in mice using established procedures [49]. The ascites fluid was partially purified by ammonium sulfate precipitation using procedures described previously [47], then lyophilized. Protein content of each of the preparations was determined using the BCA Protein Assay according to the protocols provided by the manufacturer (Thermo Fisher, Pittsburgh, PA, USA). 

### 3.6. Cross Reactivity of Mabs and Solvent Effects

The ability of isolated Mabs to cross-react with related trichothecenes was tested by CI-ELISA. Standard T2-Glc or other trichothecene mycotoxins were solubilized and diluted to 1 mg/mL with either acetonitrile or methanol. These stocks were further diluted with PBS to produce standard solutions with concentrations from 0.1 to 10,000 ng/mL. Preliminary experiments were used to establish narrower concentration ranges that were then used to determine the concentrations causing 50% inhibition of signal (IC_50_s). The CI-ELISA protocol was the same as that described for screening of the cultures, with the exception that a lower level of T2G-OVA (0.4 µg/mL) was immobilized. Because each Mab preparation contained different levels of protein, preliminary experiments were used to determine the concentrations of each of the antibodies that would provide optimal signal in the assays (an absorbance of 0.8 to 1.1 for toxin-free controls). The cell lines and corresponding protein concentrations added in the cross-reactivity tests were: 1-2 (2 µg/mL), 1-3 (4 µg/mL), 1-4 (4 µg/mL), 2-5 (4 µg/mL), 2-11 (8 µg/mL), 2-13 (2 µg/mL), 2-16 (4 µg/mL), 2-17 (4 µg/mL), 2-21 (10 µg/mL), and 2-44 (2 µg/mL). All 10 Mab were tested for cross-reactivity to T2-Glc, T-2 toxin, and HT-2 toxin. Based upon those results, further experiments were conducted to determine cross-reactivity of Mab 2-13 to 14 additional trichothecenes. 

## 4. Conclusions

Ten Mab-producing hybridoma cell lines were developed that recognized the masked mycotoxin T2-Glc as well as the parent toxin, T-2 toxin. When used in a CI-ELISA format, most of these Mabs resulted in assays with IC_50_s in the low ng/mL range, with lower cross-reactivity to HT-2 toxin. Mab from clone 2-13 was further characterized with additional cross-reactivity and solvent tolerance studies. This Mab was highly specific for T-2 toxin and T2-Glc. The region of the molecule encompassed by positions 8, 7, 15, and 4 (R1 thru R4 in Figure 1) was important for recognition. There was some flexibility in binding at position 3 (R5 in Figure 1), which was also the site of the glucose attachment. The assay with Mab 2-13 demonstrated relatively good solvent tolerance. This, combined with the ability to equally detect T-2 toxin and T2-Glc, may make this antibody useful for the simultaneous detection of both toxins in commodities. 
